# Silesaurid (Archosauria: Dinosauriformes) remains from the base of the Dockum Group (Late Triassic: Otischalkian) of Texas provide new insights to the North American record of dinosauriforms

**DOI:** 10.1002/ar.25677

**Published:** 2025-05-07

**Authors:** Frederick B. Tolchard, Brynden W. Perkins, Sterling J. Nesbitt

**Affiliations:** ^1^ Evolutionary Studies Institute University of the Witwatersrand Johannesburg South Africa; ^2^ Department of Geosciences Virginia Tech Blacksburg Virginia USA

**Keywords:** Archosauria, ontogeny, Silesauridae, Upper Triassic

## Abstract

Silesaurids (Archosauria: Dinosauriformes) are found in Middle to Upper Triassic deposits across Pangea, but few stratigraphic sections record the evolution of the group in one geographic area over millions of years. Here, we describe silesaurid remains from the oldest of the Upper Triassic stratigraphic sequence from the base of the Dockum Group, from the type locality of the Otischalkian faunachronozone. Isolated limb bones diagnostic of silesaurids include humeri, femora, and tibiae of a seemingly unique *Silesaurus*‐like taxon from the same locality (Otis Chalk Quarry 3). The femora consist of four specimens of different lengths that sample the variation of character states associated with ontogeny, also sampled previously in both silesaurids (e.g., *Asilisaurus kongwe* and *Silesaurus opolensis*) and within neotheropods within Dinosauria (e.g., *Coelophysis bauri*). Our observations of the variation in the silesaurid sample further reinforce the interpretation of high variation of morphological features common in dinosauriforms. Furthermore, we show that overpreparation of bone surfaces has hidden some of this variation in previous interpretations. The tibia growth series shows that the fibular crest of the tibia develops during ontogeny, yet another phylogenetically informative character for dinosaurs and their kin that is at least ontogenetically variable in silesaurids. The presence of silesaurids at the base of the Dockum Group (late Carnian or early Norian) conclusively shows that the group was present near the onset of deposition of Upper Triassic rocks and survived for millions of years in the same geographic area at low latitudes throughout the Late Triassic.

## 1 INTRODUCTION

Silesaurids are a group of small‐to‐medium sized dinosauriform archosaurs, with craniodental anatomy correlated with a mostly herbivorous diet (Brusatte et al., 2010; Dzik, 2003; Langer et al., 2010; Müller & Garcia, 2020; Nesbitt et al., 2010). Aspects of their anatomy, such as an anterior trochanter of the femur continuous with the femoral shaft, a weakly developed cnemial crest on the tibia, an edentulous anterior tip of the dentary, and herbivorous‐like teeth (Langer & Ferigolo, 2013; Müller & Garcia, 2020; Nesbitt et al., 2010) have led to a debated phylogenetic position among dinosauriforms. One phylogenetic hypothesis places Silesauridae as the sister taxon to Dinosauria (Cabreira et al., 2016; Langer & Ferigolo, 2013; Nesbitt, 2011) whereas the alternative hypothesis posits Silesauridae as dinosaurs within the herbivorous clade Ornithischia (Müller & Garcia, 2020). Within the “silesaurids‐as‐ornithischians” hypothesis, silesaurids either constitute part of a paraphyletic grade outside core ornithischians (Fonseca et al., 2024; Müller & Garcia, 2020, 2023; Norman et al., 2022) or as a monophyletic group as the sister taxon to the clade containing all other ornithischians (Pacheco et al., 2019). Regardless of their phylogenetic position, silesaurids are of importance to determining character optimizations in phylogenetic hypotheses, evolutionary changes among dinosaurs, and the timing of the origin of dinosaurs and closest relatives.

The fossil record of silesaurids spans much of Pangea from the Middle‐through‐Upper Triassic (Langer et al., 2010; Müller & Garcia, 2023; Nesbitt et al., 2010). The documented fossil record supports a southern Pangean origin for silesaurids given the presence of the group from the Middle Triassic (or earliest Late Triassic; Ottone et al., 2014) southern Pangaean deposits: *Gamatavus antiquus* (Pretto et al., 2022) and *Gondwanax paraisensis* (Müller, 2025) from Brazil, *Asilisaurus kongwe* from Tanzania (Nesbitt et al., 2010, 2020), and *Lutungutali sitwensis* from Zambia (Peecook et al., 2013). Silesaurid remains have been recovered worldwide from Late Triassic deposits, including those in the northern and southern parts of Pangea (Dzik, 2003; Fonseca et al., 2024; Müller & Garcia, 2020, 2023; Nesbitt et al., 2010). Within the extensive Upper Triassic deposits in western North America, silesaurids are present but seemingly rare. The Late Triassic record of silesaurids is from middle‐ and upper‐Norian deposits of North America such as the Chinle Formation (Marsh & Parker, 2020; Martz & Small, 2019; Woody et al., 2006), and the younger strata in the Dockum Group (Nesbitt et al., 2007; Nesbitt & Chatterjee, 2008; Sarıgül, 2018). However, the occurrence of silesaurids lower in the Dockum Group has only been mentioned and their anatomy and potential relationships have not been detailed.

Here, we describe silesaurid limb bones from the base of the Dockum Group, from the same locality (Otis Chalk Quarry 3) from the type fauna of the Otischalkian faunachronozone (sensu Lucas, 1998). This work represents the first formal description of this group, extending the fossil record of the silesaurids in the Dockum Group and Chinle Formation, extending their temporal range to the late Carnian or early Norian of the southwestern United States (see below). We also document the extensive variation of the femora in this small sample and hypothesize that the differences align with the patterns of ontogenetic variation in other silesaurids (Griffin & Nesbitt, 2016a) and in early diverging dinosaurs (Griffin & Nesbitt, 2016b).

## 2 METHODS AND MATERIALS

All of the bones described here were recovered from Otis Chalk Quarry 3 (Long & Murry, 1995; Stocker, 2013; Stocker et al., 2016) during excavations from 1939 to 1941 by the Works Progress Administration (Stocker, 2013). Out of the sample described here, TMM 31100‐185 (left femur), TMM 31100‐172 (left tibia), and TMM 31100‐1319 (right humerus) were prepared soon after recovery using chisels and grinding tools to remove the red mudstone and calcite adhering to the surfaces. The use of grinding tools, consequently, resulted in artificially smoothing of surfaces, removal of fine features, and exposure of parts of the inner surfaces of the bone. This combination of damage resulted in the misidentification of one of the bones (TMM 31100‐185) as a pseudosuchian (i.e., ornithosuchid, Long & Murry, 1995). In contrast, all of the other bones in our study (i.e., TMM 31100‐1303, TMM 31100‐1304, TMM 31100‐1309, TMM 31100‐1311, and TMM 31100‐1330) were identified in the TMM collections unprepared and in their original excavated state. These specimens were then prepared using modern methods in attempts to preserve all of the delicate bone scars and original surfaces of the bones. Matrix was removed by carbide needles and a Microjack 1 (www.paleotools.com) by S.J.N.

To determine phylogenetic affinities of the Otis Chalk silesaurid, we scored phylogenetic characters for the humerus, femora, and the most complete tibia (TMM 31100‐172) into the matrix of Müller (2025; Supp 1), which is the most recent iteration of the matrix of Norman et al. (2022). The characters were scored into the matrix using Mesquite (version 3.81; Maddison & Maddison, 2023). We also scored characters for a combined operational taxonomic unit (OTU) consisting of the humerus, tibia (TMM 31100‐172), and the femur determined to represent the most mature individual (TMM 31100‐1303; see below). We ran four sets of phylogenetic analyses: one using the combined OTU, and three separate analyses for each of the following femora: TMM 31100‐1309; TMM 31100‐1303; and TMM 31100‐1304. We ran all analyses in TNT (Goloboff & Catalano, 2016) using new technology search algorithms, followed by a round of tree bisection and reconstruction (TBR) swapping on each analysis, following the methods and analysis of Müller (2025).

To assess ontogenetic variation within the silesaurid sample, we scored bone scar and other characters associated with ontogenetic change of each femoral specimen following the characters of Griffin and Nesbitt (2016a) (Table 1). We used PAUP* (v. 4.0b10; Swofford, 2002) to run a heuristic search with the tree‐bisection‐reconnection algorithm, running 20,000 replicates with the least mature semaphoront (TMM 31100‐1309, see Section 8) as the outgroup. We then collapsed any zero‐length branches on the trees recovered. Our analysis resulted in two most parsimonious trees (MPTs). The strict consensus resulted in a polytomy between the three more‐mature semaphoronts.

**TABLE 1:** Bone scar information and observed maturity score of the femora presented in this sample.

Specimen	(1) dltp	(2) cfb	(3) dlp	(4) als	(5) dlta	(6) ts	(7) at	(8) at+ts	(9) lia	(10) lip	(11) 4th	Observed maturity score
TMM 31100‐1303	1	0	1	0	2	1	1	1	1	1	1	10
TMM 31100‐1304	1	1	1	1	1	1	1	0	0	1	1	9
TMM 31100‐1309	0	0	0	0	1	0	1	0	0	1	0	3
TMM 31100‐185	?	?	0	?	?	0	1	0	?	?	1	NA

*Note*: (1) Femur, proximal portion, posteromedial surface, scar on the posterior portion of the dorsolateral trochanter (dltp): absent (0); present (1). (2) Femur, proximal portion, posterolateral edge, M. caudofemoralis brevis insertion scar (cfb): absent (0); present (1). (3) Femur, proximal portion, medial surface, distolateral protrusion on the fourth trochanter (dlp): absent (0); present (1). (4) Femur, proximal portion, anterolateral surface, anterolateral scar (als): absent (0); present (1). (5) Femur, proximal portion, anterolateral surface, anterior portion of the dorsolateral trochanter (dlta): absent (0); present and sharp (1); present and rounded (2). (6) Femur, proximal portion, anterolateral surface, trochanteric shelf (ts): absent (0); present (1). (7) Femur, proximal portion, anterolateral surface, anterior trochanter (at): absent (0); present (1). (8) Femur, proximal portion, anterolateral surface, anterior trochanter and trochanteric shelf (at + ts) continuous: absent (0); present (1). (9) Femur, proximal portion, anteromedial edge, linea intermuscularis cranialis (lia): absent (0); present (1). (10) Femur, proximal portion, posterolateral edge, linea intermuscularis caudalis (lip): absent (0); present (1). (11) Fourth trocahnter (4th), form: Ridge (0); rounded (1). Observed maturity score is the sum of all character transformations (states 1 and 2) that have occurred.

## 3 INSTITUTIONAL ABBREVIATIONS

CAPPA/UFSM: Centro de Apoio à Pesquisa Paleontológica da Quarta Colônia da Universidade Federal de Santa Maria, Rio Grande do Sul, Brazil; DMNH: Denver Museum of Nature and Science, Colorado, USA; MCN PV: Museu de Ciências Naturais, Fundação Zoobotânica do Rio Grande do Sul, Rio Grande do Sul, Brazil; Paleovertebrates Collection: NMMNH, New Mexico Museum of Natural History and Science, New Mexico, USA; NMT: National Museum of Tanzania, Dar es Salaam, Tanzania; PULR‐V: Museo de Ciencias Antropológicas y Naturales, Colección Paleontológica de Vertebrados, Universidad Nacional de La Rioja, La Rioja, Argentina; TMM: Collections at the Vertebrate Paleontology Laboratory (former Texas Memorial Museum), University of Texas at Austin, Texas, USA; ZPAL: Institute of Paleobiology of the Polish Academy of Sciences, Warsaw, Poland.

## 4 GEOLOGICAL SETTING

**FIGURE 1:**
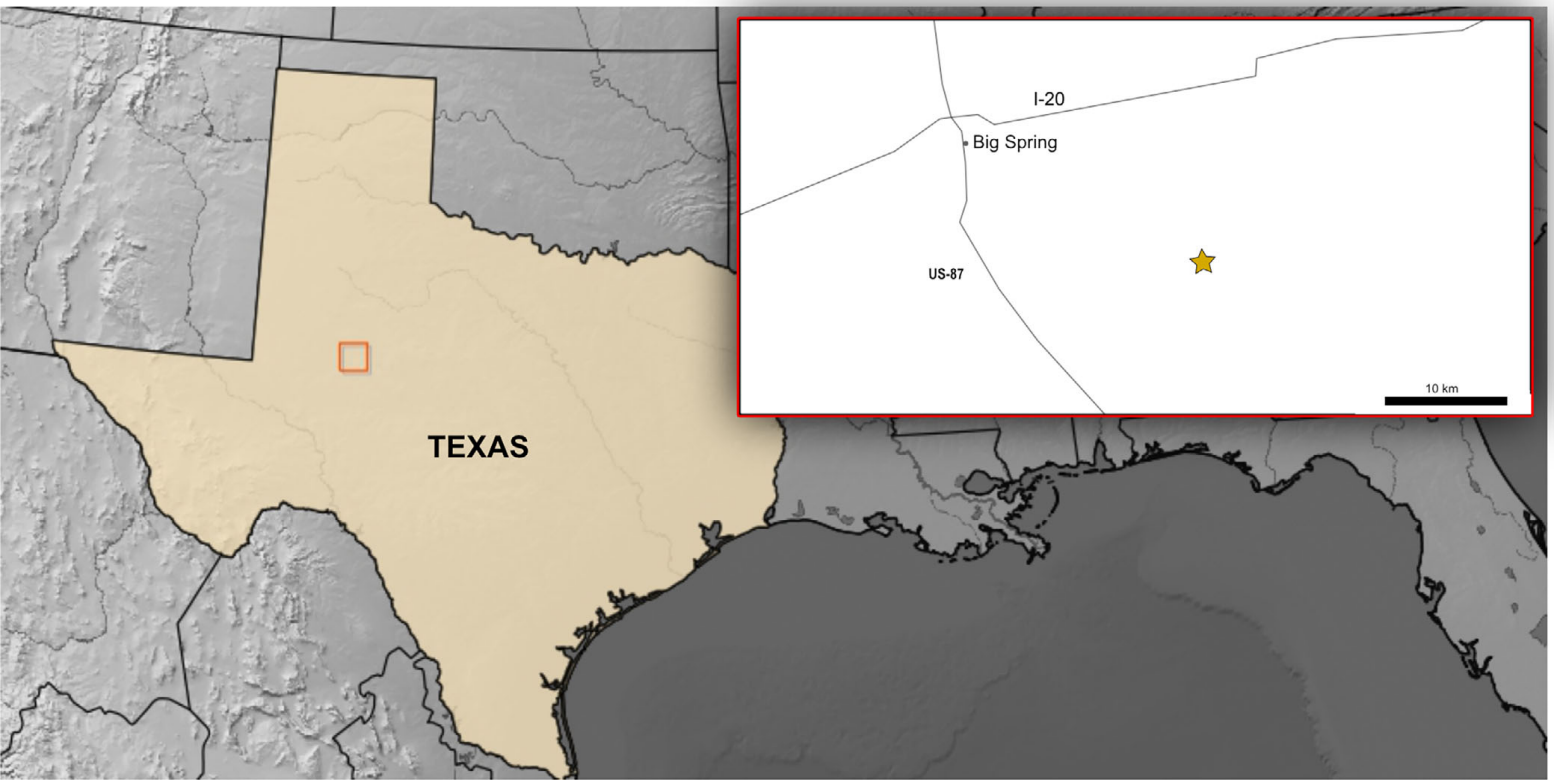
Map showing locality within Texas. Location of inset indicated by red box, with location of Otis Chalk assemblage Q3 indicated with a star. Scale bar equals 10 km.

The Otis Chalk localities (Figure 1) are a set of highly fossiliferous horizons of Upper Triassic strata at the base of the Dockum Group (Lucas et al., 1993; Martz, 2008; Stocker, 2013; To et al., 2021). In lieu of radioisotopic data, the Otis Chalk exposures have been estimated as late‐Carnian to early‐Norian in age based on biostratigraphic correlation between vertebrate fossils recovered from Otis Chalk deposits and those in other Dockum Group localities, those in the Chinle Formation (Stocker, 2013) and across other Upper Triassic localities (see Lucas, 1998). Archosauromorph taxa documented from the Otis Chalk localities include allokotosaurs (Nesbitt et al., 2022), phytosaurs (Lucas et al., 2002), aetosaurs (Hunt & Lucas, 1990), poposauroid pseudosuchians (Weinbaum & Hungerbühler, 2007), early‐branching crocodylomorphs (To et al., 2021), and theropod dinosaurs (Nesbitt & Ezcurra, 2015).

## 5 SYSTEMATIC PALEONTOLOGY

Archosauria Cope (1869) sensu Gauthier et al. (1985).

Dinosauriformes Benton, 1985 (sensu Ezcurra et al., 2020).

Silesauridae Langer et al. (2010), Nesbitt et al. (2010).

Referred material: Right humerus, TMM 31100‐1319; two left femora, TMM 31100‐1303, TMM 31100‐185; two right femora, TMM 31100‐1304, TMM 31100‐1309; two fragmentary right tibiae, TMM 31100‐1311, TMM 31100‐1330; one complete left tibia, TMM 31100‐172.

Locality: Otis Chalk Quarry 3 (TMM 31100), Howard County, Texas, USA. Dockum Group; latest Carnian–early Norian, Upper Triassic (Martz, 2008; Martz & Parker, 2017; Stocker, 2013; Stocker et al., 2016). All specimens were excavated from the same quarry at the same horizon.

Rationale for taxonomic assignment: We refer a total of seven specimens to Silesauridae based on several lines of evidence.

**FIGURE 2:**
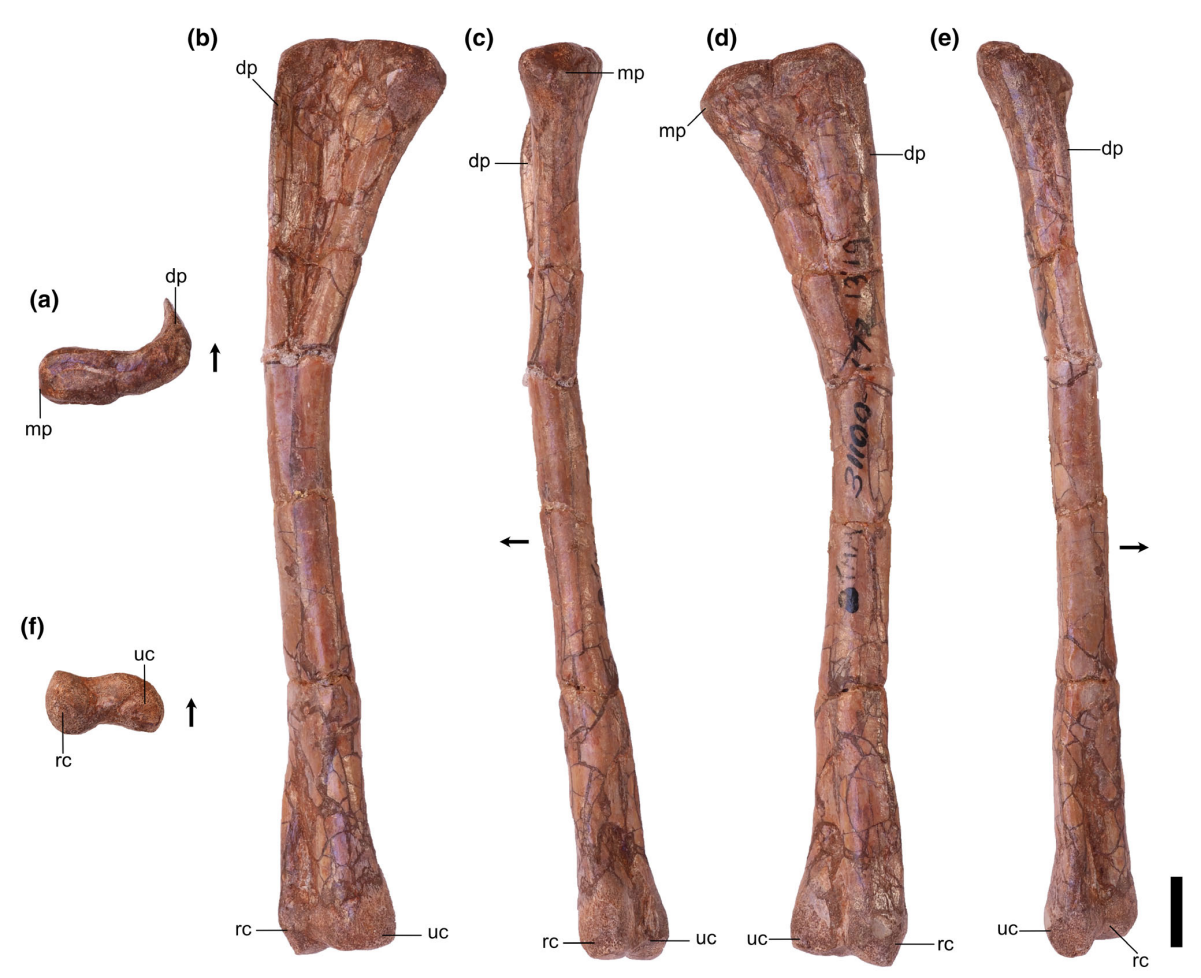
Silesauridae (TMM 31100‐1319) right humerus in proximal (a), anterior (b), medial (c), posterior (d), lateral (e), and distal (f) views. Dp, deltopectoral crest; mp, medial process; rc, radial condyle; uc, ulnar condyle. Arrows represent anterior direction. Scale bar equals 1 cm.

We tentatively identify humerus TMM 31100‐1319 (Figure 2) as a silesaurid based on comparative anatomy. The medial crest is weakly developed and extends <20% the dorsoventral length of the humerus, similar to, for example, that of *Silesaurus opolensis* (e.g., ZPAL AbIII/452; Piechowski & Talanda, 2020). Furthermore, the proximal head of the humerus is mediolaterally expanded relative to the midshaft (>2:1), another similarity with *Silesaurus opolensis* (e.g., ZPAL AbIII/452; Piechowski & Talanda, 2020) that differentiates TMM 31100‐1319 from other contemporaneous archosauriforms (e.g., *Shuvosaurus inexpectatus*; Nesbitt, 2011; Nesbitt & Chatterjee, 2024). Because there are no currently identified phylogenetically tested character states of the humerus that represent silesaurid apomorphies, we can only make this diagnosis on the basis of a broad morphological similarity between TMM 31100‐1319 and the humerus of *Silesaurus opolensis* (Piechowski & Tałanda, 2020). The size of the humerus is consistent with the sizes of the femora when compared to *Silesaurus opolensis* (Dzik, 2003).

All four femora (TMM 31100‐185, TMM 31100‐1303, TMM 31100‐1304, and TMM 31100‐1309) are referable to Silesauridae based on a conspicuous notch ventral to the proximal head of the femur (Nesbitt, 2011; Character 304.1), which is apomorphic of Silesauridae when the clade is found as monophyletic (Nesbitt et al., 2010). TMM 31100‐1309 is morphologically distinct from the others because it lacks a posteromedial tuber on the posterior surface (Norman et al., 2022; Character 205.2). Among silesaurids, TMM 31100‐1309 shares this character state with *Lutungutali sitwensis* and *Kwanasaurus williamparkeri* (Norman et al., 2022). We do not assert TMM 31100‐1309 to represent a different taxon from the other specimens, as this difference in shape is likely a result of ontogenetic variation (see Section 8). From the perspective of comparative anatomy, the longer and likely more mature specimens (TMM 31100‐1303, TMM 31100‐1304, and TMM 31100‐185) are most similar to *Silesaurus opolensis* and *Sacisaurus agudoensis* (see Section 6).

We refer to the tibiae TMM 31100‐1311, TMM 31100‐1330, and TMM 31100‐172 based on comparative anatomy and a suite of shared characters with other silesaurids, but that exclude them from other later‐branching dinosauriforms: a posterolateral flange on the distal portion of the tibia that extends well lateral to the anterolateral corner (Norman et al., 2022; Character 239.2); proximal condyles of the tibia separated by a shallow notch (Norman et al., 2022; Character 237.0); and a rounded posteromedial surface of the distal portion of the tibia (Norman et al., 2022; Character 242.0).

## 6 ANATOMICAL DESCRIPTION

### 6.1 Humerus

Overview: A single, complete right humerus (TMM 31100‐1319) was identified (Figure 2). Extensive abrasion from the original preparation and some crushing obscure many of the finer anatomical details. The humerus is nearly straight proximodistally in medial and lateral views, and the dorsal margin is nearly flat in anterior and posterior views (Figure 2). In anterior view, the medial margin flares proximally. The lateral margin is nearly straight in anterior view, given that the deltopectoral crest does not extend far laterally. On the distal margin, neither the radial nor ulnar condyle projects distally, though this may be distorted by abrasion (Figure 2). Neither the proximal nor distal portions flare mediolaterally beyond the shaft to any great extent. Overall, the shape and character states of TMM 31100‐1319 look most similar to *Silesaurus opolensis* (e.g., ZPAL AbIII/452; Piechowski & Tałanda, 2020) and to CAPPA/UFSM 0397 (“possible silesaurid” humerus material from the Santa Maria Supersequence—*Hyperodapedon* AZ, Early Norian) of Brazil (Doering et al., 2024). It also bears resemblance to the pseudosuchian *Shuvosaurus inexpectatus* (Nesbitt, 2011; fig. 31) by having a low deltopectoral crest and a near (dorsoventrally) straight lateral margin. However, the dorsal margin of the humerus of *Shuvosaurus inexpectatus* (Nesbitt, 2011; fig. 31) in anterior view is far more convex than in either TMM 3110‐1319 or *Silesaurus opolensis*. Additionally, the mediolateral length of the proximal portion of the humerus expands to well over double the mediolateral diameter of the humeral shaft midway (character 236, state 0; Nesbitt, 2011) in TMM 31100‐1319, whereas this ratio is less than 2:1 in *Shuvosaurus inexpectatus* (character 236, state 1; Nesbitt, 2011). In general, TMM 31100‐1319 shares identical scorings for characters of the humerus of *Silesaurus opolensis* in the character matrix of Nesbitt (2011) (230.0, 231.0, 232.0, 233.0, 234.1, 235.0, and 236.0) but this is insufficient to diagnose TMM 31100‐1319 as a silesaurid because this suite of characters represents the plesiomorphic archosaurian condition and is shared by, for example, *Vancleavea campi* and *Euparkeria capensis* (Nesbitt, 2011). As a result, we are only able to assign this humerus tentatively to Silesauridae based on similarity to *Silesaurus opolensis*.

Proximal portion: The posterior margin of the humerus is convex in proximal view. The humeral head shape is obscured by abrasion but likely does not extend beyond the posterior margin of the dorsal surface of the humerus. The lateral portion of the anterior margin is concave between the deltopectoral crest and the medial process. The medial process is a small, rounded margin on the medial side of the proximal surface. The proximal margin of the deltopectoral crest curves and tapers to a point a short way directly anteriorly. On the lateral margin of the humerus, the deltopectoral crest is a thin, blade‐like ridge that extends anteriorly (Figure 2). The deltopectoral crest is present for a little less than 15% of the proximodistal length of the humerus, which is shorter than other silesaurids like *Asilisaurus kongwe* (20%; Nesbitt et al., 2020). It shares this shape with the potential silesaurid CAPPA/UFSM 0397 (Doering et al., 2024). On the anterior surface, the area between the deltopectoral crest and the medial process forms a concave surface representing the attachment area for m. coracobrachialis brevis (Piechowski, 2021). Much of the finer detail on the posterior surface is obscured by weathering.

The morphology of the proximal portion of the shaft is most similar to that of *Silesaurus opolensis* (Dzik, 2003; Figure 9). In particular, the shape and length of the deltopectoral crest are similar to *Silesaurus opolensis* (e.g., ZPAL AbIII/452; Piechowski & Talanda, 2020) and *Kwanasaurus williamparkeri* (e.g., DMNH EPV.59302; Martz & Small, 2019).

Distal portion: The distal end of the humerus is less mediolaterally expanded than the proximal end (see Table 2). Both the ulnar (medial) and radial (lateral) condyles have rounded distal margins. The radial condyle is smaller than the ulnar condyle, and its long axis is oriented anteroposteriorly, and the anterior portion of the condyle is slightly deflected laterally. The long axis of the ulnar condyle is oriented mediolaterally and deflected anteromedially. Both condyles extend equally far distally, though there is a lot of abrasion on the ventral surface likely obscuring the original shape of the distal margin of the humerus (Figure 2). Given the extent of abrasion on the distal portion, it is difficult to make clear comparisons, but the overall morphology of the distal portion of the humerus is nearly identical to that of *Silesaurus opolensis* (e.g., ZPAL AbIII/452; Piechowski & Talanda, 2020).

**TABLE 2:** Measurements of the long bone elements presented in this sample.

	TL	PL	PW	DL	DW	MW	Circ.
Humerus							
TMM 31100‐1319	115	22	8	15	7	8	29
Femora							
TMM 31100‐1303	152	25	12	25	24	16	43
TMM 31100‐1304	155	29	13	30	22	15^a^	52
TMM 31100‐1309	NA	19	9	NA	NA	11	38
TMM 31100‐185	141^a^	22	10^a^	25	19	15	48
Tibiae							
TMM 31100‐172	116	14^a^	29^a^	9^a^	18^a^	10^a^	35
TMM 31100‐1311	NA	16	28	12	19	NA	NA
TMM 31100‐1330	112^a^	11	17	7	12	6	25

*Note*: Circ., circumference at midshaft; DW, distal width (anteroposterior); DL, distal length (mediolateral); PL, proximal length (mediolateral); PW, proximal width (anteroposterior); MD, midshaft diameter; TL, total length; NA, not available. TMM 31100‐1309 length estimated using Griffin and Nesbitt (2016a) equation (4.69*x* + 9.19; where ‘*x*’ is the proximal width): 98 mm. Maximum preserved length: 74 mm.

^a^ Measurement unreliable due to distortion or breakage.

### 6.2 Femora

**FIGURE 3:**
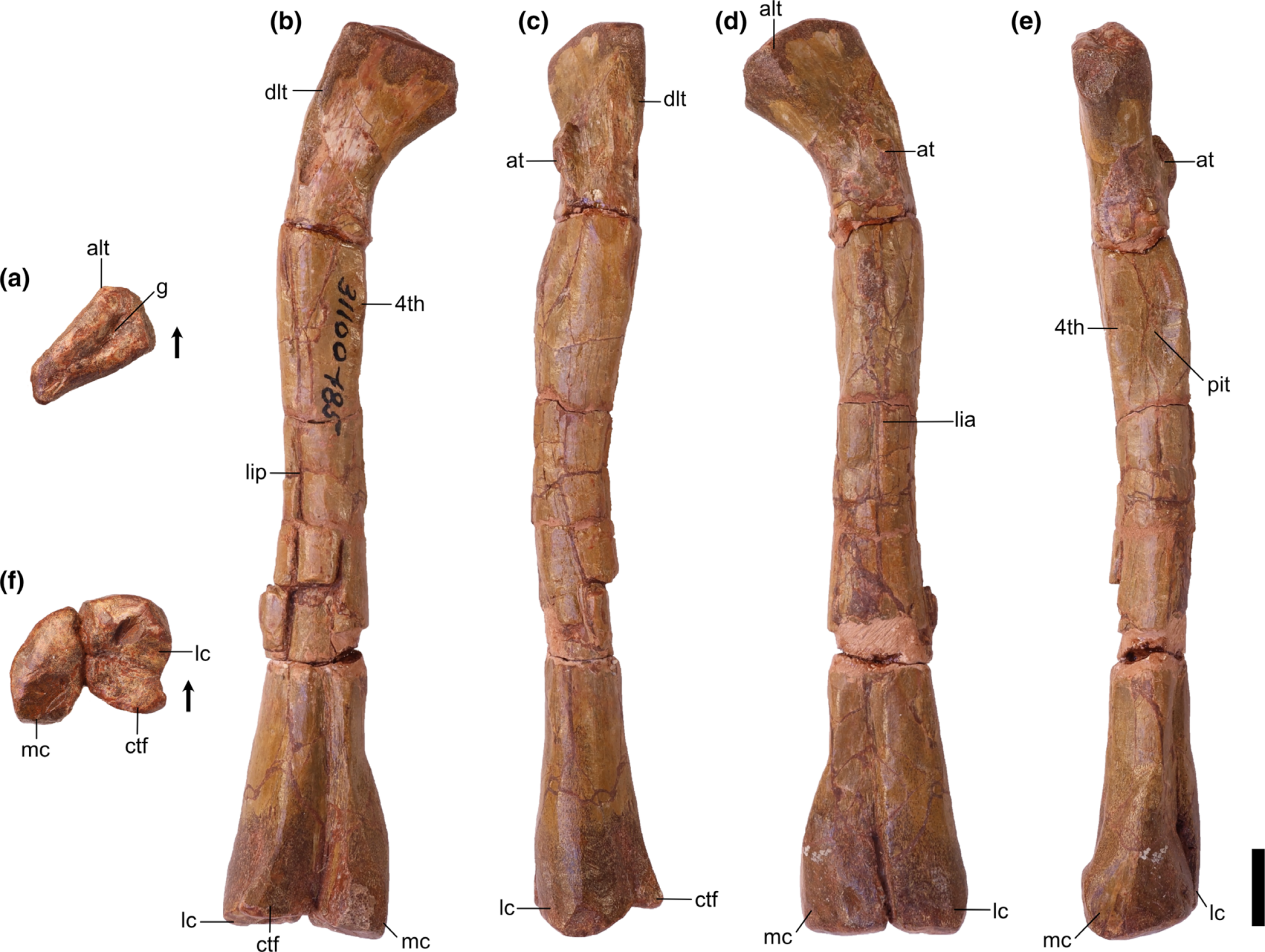
Silesauridae (TMM 31100‐185) left femur in proximal (a), posteromedial (b), posterolateral (c), anterolateral (d), anteromedial (e), and distal (f) views. 4th, fourth trochanter; alt, anterolateral tuber; at, anterior trochanter; ctf, crista tibiofibularis; dlt, dorsolateral trochanter; g, groove; lc, lateral condyle; lia, linea intermuscularis cranialis; lip, linea intermuscularis caudalis; mc, medial condyle; pit, pit. Arrows represent anterior direction. Scale bar equals 1 cm.

**FIGURE 4:**
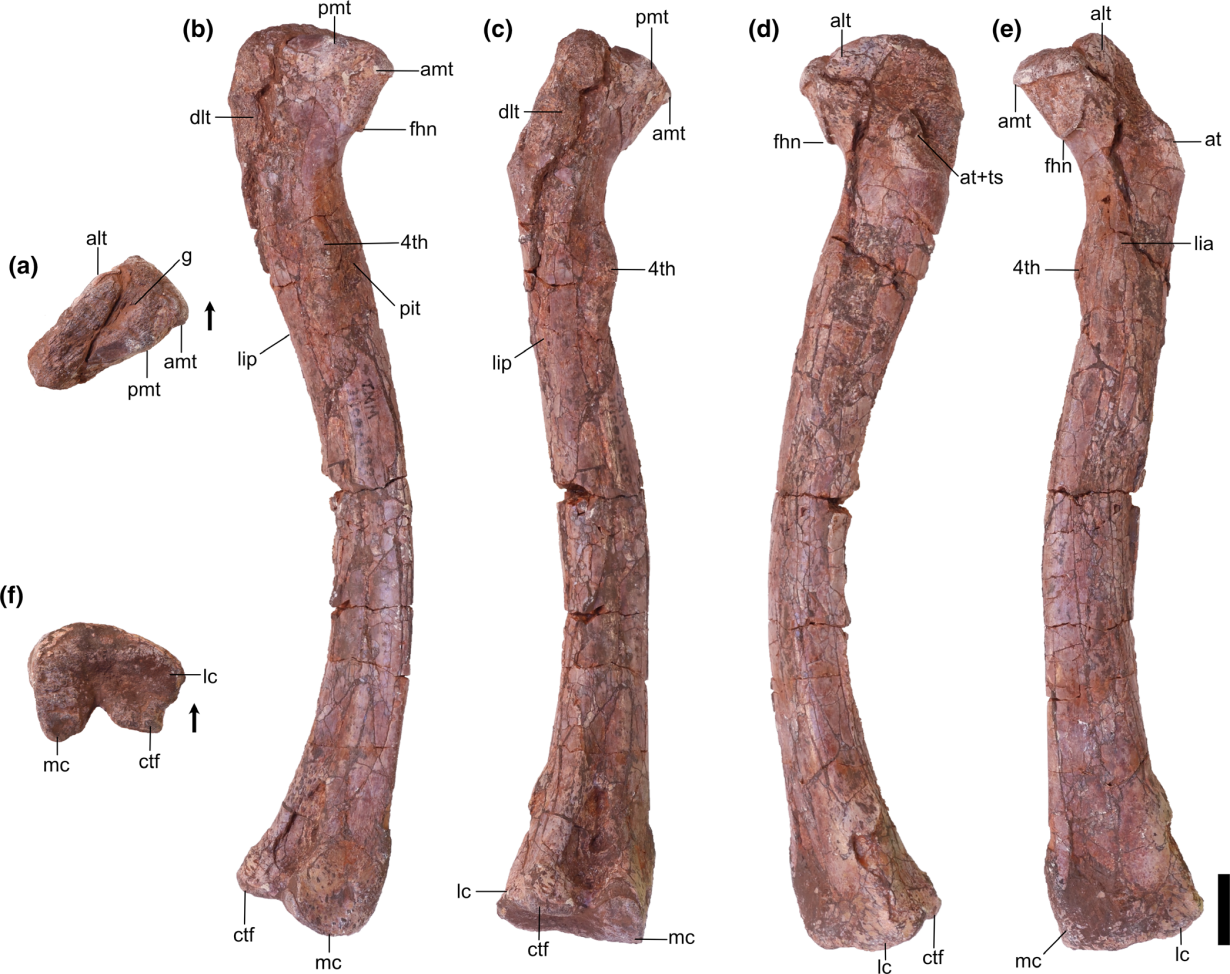
Silesauridae (TMM 31100‐1303) left femur in proximal (a), posteromedial (b), posterolateral (c), anterolateral (d), anteromedial (e), and distal (f) views. 4th, fourth trochanter; alt, anterolateral tuber; amt, anteromedial tuber; at, anterior trochanter; ctf, crista tibiofibularis; dlt, dorsolateral trochanter; fhn, femoral head notch; g, groove; lc, lateral condyle; lia, linea intermuscularis cranialis; lip, linea intermuscularis caudalis; mc, medial condyle; pit, pit; pmt, posteromedial tuber; ts, trochanteric shelf. Arrows represent anterior direction. Scale bar equals 1 cm.

**FIGURE 5:**
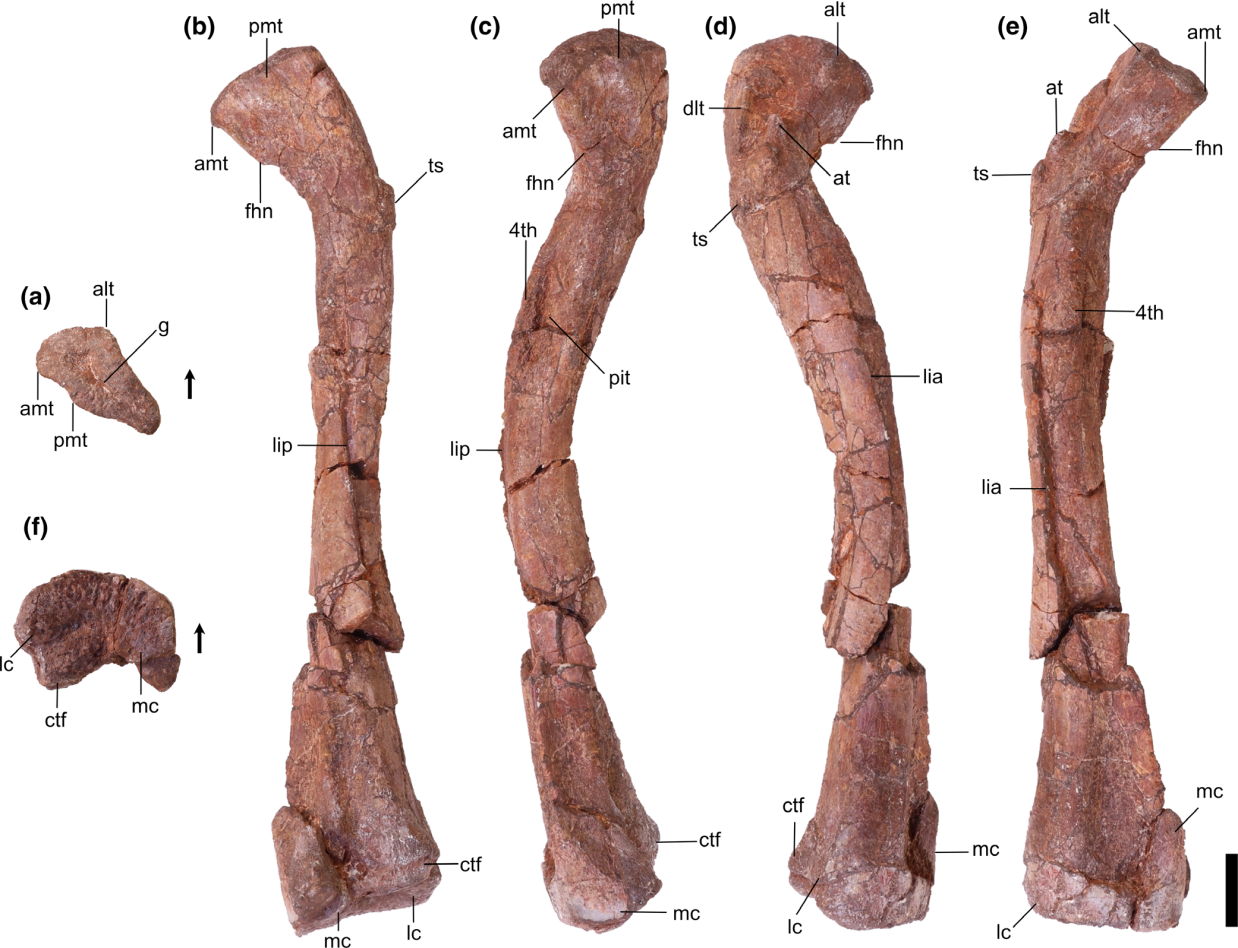
Silesauridae (TMM 31100‐1304) right femur in proximal (a), posteromedial (b), posterolateral (c), anterolateral (d), anteromedial (e), and distal (f) views. 4th, fourth trochanter; alt, anterolateral tuber; amt, anteromedial tuber; at, anterior trochanter; ctf, crista tibiofibularis; dlt, dorsolateral trochanter; fhn, femoral head notch; g, groove; lc, lateral condyle; lia, linea intermuscularis cranialis; lip, linea intermuscularis caudalis; mc, medial condyle; pit, pit; pmt, posteromedial tuber; ts, trochanteric shelf. Arrows represent anterior direction. Scale bar equals 1 cm.

**FIGURE 6:**
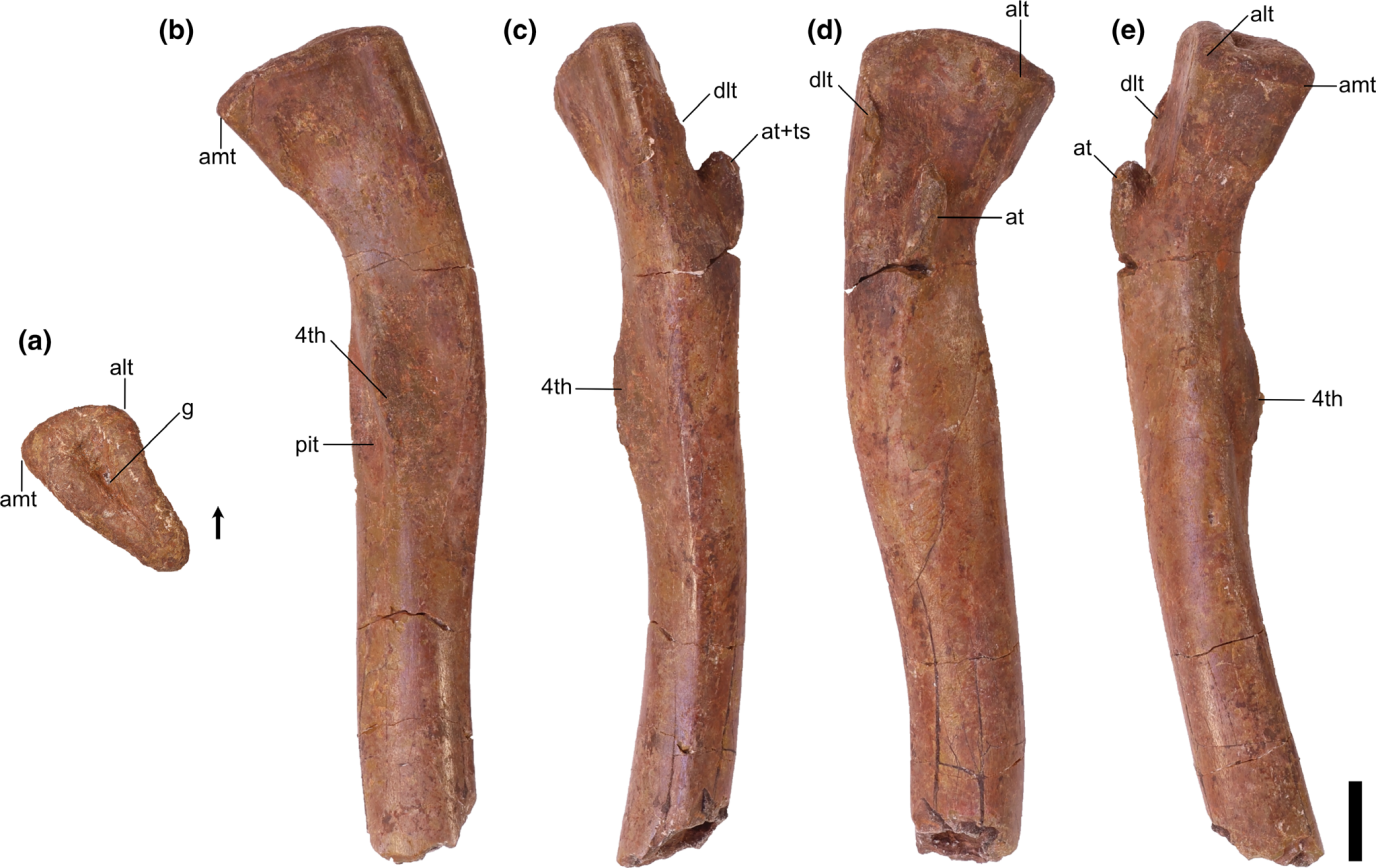
Silesauridae (TMM 31100‐1309) right femur in proximal (a), posteromedial (b), posterolateral (c), anterolateral (d), and anteromedial (e) views. 4th, fourth trochanter; alt, anterolateral tuber; amt, anteromedial tuber; at, anterior trochanter; dlt, dorsolateral trochanter; g, groove; pit, pit. Arrows represent anterior direction. Scale bar equals 1 cm.

**FIGURE 7:**
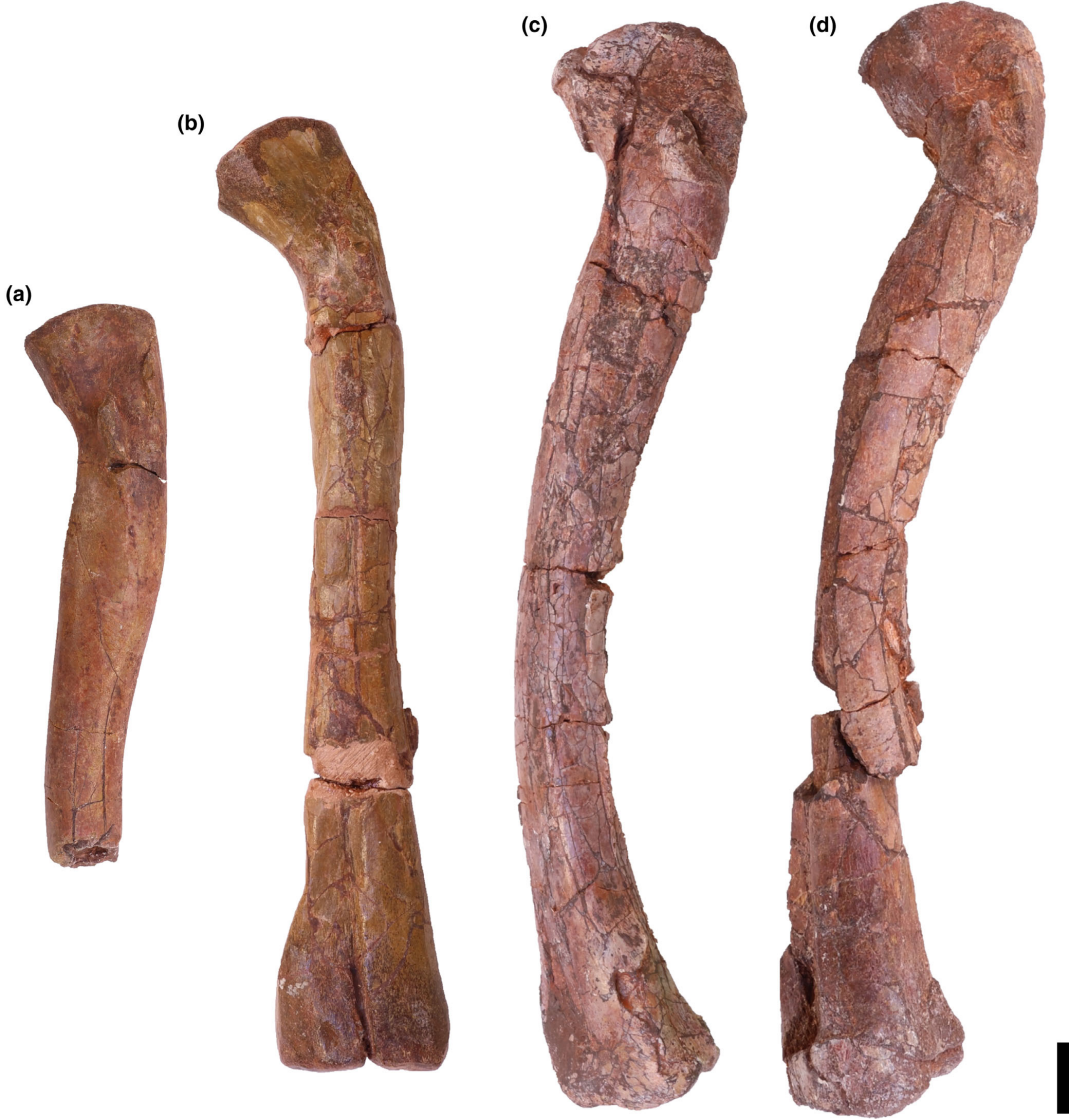
Silesauridae femora TMM 31100‐1309 (a), TMM 31100‐185 (b), TMM 31100‐1303 (c), and TMM 31100‐1304 (d) in anterolateral view. (a) and (d) are mirrored. Scale bar equals 1 cm.

Overview: There are two left (TMM 31100‐185, Figure 3; TMM 31100‐1303, Figure 4) and two right (TMM 31100‐1304, Figure 5; TMM 31100‐1309, Figure 6) femora (Figure 7). All specimens except TMM 31100‐1309 are complete (the latter is missing its distal half). The preserved portion of TMM 3110‐1309 is well preserved, with most features clearly discernible (Figure 6). The entire surfaces of TMM 31100‐1303 and TMM 31100‐1304 are covered in cracks and breaks obscuring some finer details, though the larger features are mostly visible (Figures 4 and 5) whereas the surface of TMM 31100‐185 is artificially smoothed from the preparation process (Figure 3) and the bone has incurred numerous breaks and is held together with plaster and an installed metal rod in its center. The shafts of TMM 31100‐1303 and TMM 31100‐1304 are sigmoidal in lateral and anterior views, similar to *Silesaurus opolensis* (Dzik, 2003), *Sacisaurus agudoensis* (Langer & Ferigolo, 2013), *Asilisaurus kongwe* (Nesbitt et al., 2010), and *Eucoelophysis baldwini* (Ezcurra, 2006). TMM 31100‐185 is dorsoventrally straight in all views, though this is an artifact of preparation and does not reflect the original shape. TMM 31100‐1303 and TMM 31100‐1304 are larger specimens of similar size class, and TMM 31100‐1309 and TMM 31100‐185 are smaller (Table 2).

Proximal portion: The long axis of the femoral head is oriented anteromedially to posterolaterally. The long axis of the proximal surface is marked by a deep groove, similar to the condition in *Silesaurus opolensis* (e.g., ZPAL Ab III/361/20; Dzik, 2003), and *Asilisaurus kongwe* (Nesbitt et al., 2020). The anterior margin of the proximal surface extends anteromedially with a slight posterior curve to the anterolateral tuber. The medial margin is gently concave in proximal view and extends posteromedially to the anteromedial tuber. In TMM 31100‐1303, the medial margin is straighter than in the other specimens, and the anterior tubera are on a similar anteroposterior plane (Figure 4). In all specimens, the anteromedial and anterolateral tubera are pronounced. The posterior margin of the proximal surface is straight mediodistally.

The posteromedial tuber is a rounded protuberance located just posterior to the anteromedial tuber. The margin between the antero‐ and posteromedial tubera, formed by the ligament sulcus, is concave; the lack of a clear gap appears to be an effect of preservation. The posteromedial tuber is less pronounced than the anteromedial tuber in the larger specimens, contrasting with *Gamatavus antiquus* (Pretto et al., 2022) where the posteromedial tuber is the largest. The posteromedial tuber is absent in TMM 31100‐1309, like in *Amanasaurus nesbitti* (Müller & Garcia, 2023), *Diodorus scytobrachion* (Kammerer et al., 2011), and *Sacisaurus agudoensis* (Langer & Ferigolo, 2013). It is possible that the development of this tuber is ontogenetically variable in dinosauriform taxa (see Ontogeny section).

The dorsolateral trochanter is present in all specimens as a raised sheet of bone along the dorsolateral margin of the femoral head. On TMM 31100‐1303 (Figure 4), it forms a raised scar with a coarse texture. The anterior portion of the dorsolateral trochanter extends proximomedially from the posteroventralmost margin of the head of the femur. The posterior portion extends a short way distomedially along the posterior surface of the femur as a scar. The condition of the dorsolateral trochanter in TMM 31100‐1303 is similar to the condition expressed in NMT RB169 (*Asilisaurus kongwe*). In TMM 31100‐1304, the anterior portion of the dorsolateral trochanter is a prominent, bladelike protrusion of bone (Figure 5) whereas the posterior portion, if present, is obscured by breakage. In TMM 31100‐1304, the dorsolateral trochanter is similar to illustrated specimens of *Sacisaurus agudoensis* (e.g., MCN PV10014; Langer & Ferigolo, 2013) and *Lewisuchus admixtus* (e.g., PULR V‐111; Agnolin et al., 2022). The posterior scars on the dorsolateral trochanter (as per Griffin & Nesbitt, 2016a) are not visibly present on TMM 31100‐185 (Figure 3) and TMM 31100‐1309 (Figure 6). In each case, the anterior portion of the dorsolateral trochanter is present as a modest blade‐like ridge. *Asilisaurus kongwe* is also hypothesized to show an ontogenetic pattern wherein the dorsolateral trochanter changes through stages from a modest ridge on the anterior margin to a scar extending posteriorly and further anteriorly (Griffin & Nesbitt, 2016a).

The anterolateral scar, as per Griffin & Nesbitt (2016a), is present on TMM 31100‐1304 as a small subcircular growth of bone situated directly medially to the dorsalmost extent of the dorsolateral trochanter (Figure 5). In this specimen, the scar resembles that of *Asilisaurus kongwe* (NMT RB159; Griffin & Nesbitt, 2016a). The scar is visible on TMM 31100‐1303 as a shallow concavity ventral to approximately the mediolateral midway of the anterodorsal margin of the femoral head. The anterolateral scar is not present on TMM 31100‐185 (Figure 3) or TMM 31100‐1309 (Figure 6).

The anterior trochanter and trochanteric shelf differ among the specimens. In TMM 31100‐185 and TMM 31100‐1309, the form of the anterior trochanter is a thin, proximally straight, blade‐like ridge extending distally from directly distal to the mediolateral midpoint of the anterior surface of the femoral head (Figures 3 and 6). The anterior trochanter in these specimens is well separated from the shaft of the femur by a marked cleft (Müller & Garcia, 2023). In TMM 31100‐1309, there is a conspicuous hook‐like projection extending proximally from the trochanter (Figure 6). This same feature may be present in TMM 31100‐185, but this is damaged. The shape of the anterior trochanter of these smaller specimens bears a strong resemblance to the femora of *Kwanasaurus williamparkeri* (e.g., DMNH EPV.125924; Martz & Small, 2019) and *Amanasaurus nesbitti* (CAPPA/UFSM 0374). It is also similar to those of *Eucoelophysis baldwini* (e.g., NMMNH P‐22298; Sullivan & Lucas, 1999), *Sacisaurus agudoensis* (e.g., MCN PV10014; Langer & Ferigolo, 2013), some specimens assigned to *Silesaurus opolensis* (e.g., ZPAL AbIII/361/23; Piechowski & Tałanda, 2020), and some smaller specimens of *Asilisaurus kongwe* (e.g., NMT RB169). Neither TMM 31100‐185 nor TMM 31100‐1309 have trochanteric shelves.

On the larger specimens (TMM 31100‐1303 and TMM 31100‐1304), the anterior trochanter is a curved, bulbous ridge that extends distally and laterally from the area distal to the femoral head (Figures 4 and 5). On each of the larger specimens, the anterior trochanter is seamlessly connected to the trochanteric shelf, which extends mediolaterally and terminates along the posterolateral margin of the femur. The trochanter is also connected and ossified to the shaft of the femur, contrasting with the smaller specimens. In TMM 31100‐1304, the proximal margin of the anterior trochanter and trochanteric shelf is nearly flat, and the lateral margin is sharp in proximal view. In TMM 31100‐1303, that same surface appears more rounded, though this may be the result of breakage. This latter condition is similar to that of some *Asilisaurus kongwe* specimens (e.g., NMT RB169, NMT RB221; Griffin & Nesbitt, 2016a), *Lewisuchus admixtus* (PULR V‐111; Agnolín et al., 2022), and some *Silesaurus opolensis* specimens (e.g., ZPAL AbIII/361/21; Piechowski et al., 2014).

The anterior portion of the linea intermuscularis cranialis is only visibly present in TMM 31100‐1303. In this specimen, the linea intermuscularis cranialis consists of two parallel raised ridges oriented proximodistally, which extending distally 30% of the length of the femur. This ‘double‐ridged’ feature is also present in other silesaurids (e.g., *Asilisaurus kongwe*, NMT RB159).

The shape of the fourth trochanter differs as two distinct morphotypes across the specimens. The fourth trochanter in TMM 31100‐1309 is a sharp, mediodistally thin ridge (Figure 6). The ridge extends proximodistally along the medial side of the proximal portion of the shaft of the femur distal to the femoral head. It is mostly straight in posterior view, though the posterior margin does curve medially around the dorsoventral middle of the trochanter. The posterior margin of the trochanter is flat in anteromedial view. The fourth trochanter characteristics of TMM 31100‐1309 are most similar to *Gamatavus antiquus* (UFSM 11348b, Pretto et al., 2022), *Sacisaurus agudoensis* (e.g., MCN PV10014; Langer & Ferigolo, 2013) and also similar to specimens assigned to *Asilisaurus kongwe* (e.g., NMT RB221; Griffin & Nesbitt, 2016a) and *Silesaurus opolensis* (e.g., ZPAL AbIII/361/21; Piechowski et al., 2014). The medial margin of the trochanter hangs over a deep cavity or pit representing the insertion scar for M. caudofemoralis longus. This pit is present and of similar aspect in numerous dinosauriform taxa, such as *Sacisaurus agudoensis* (e.g., MCN PV10014; Langer & Ferigolo, 2013) and *Asilisaurus kongwe* (e.g., NMT RB159).

On the other hand, the fourth trochanter in TMM 31100‐1303 is characterized by a bulbous, mediolaterally thick ridge (Figure 4). The ventral portion of the trochanter extends farther laterally than TMM 31100‐1309. A subtle distolateral protrusion (as per Griffin & Nesbitt, 2016a) can be found at the distalmost part of the trochanter. The pit medial to the trochanter is present, but it is not as pronounced as in TMM 31100‐1309. Collectively, this morphological suite of the fourth trochanter is similar to those of certain specimens of *Asilisaurus kongwe* (e.g., NMT RB159), *Gondwanax paraisensis* (CAPPA/UFSM 0417; Müller, 2025), and *Silesaurus opolensis* (e.g., ZPAL AbIII/361/23; Piechowski et al., 2014).

In TMM 31100‐185, the fourth trochanter is artificially abraded during preparation and is laterally deflected beyond its natural condition (Figure 3). In TMM 31100‐1304, the fourth trochanter is also obscured by breakage and surface weathering.

The M. caudofemoralis brevis attachment scar is only discernible in TMM 31100‐1304. The M. caudofemoralis brevis attachment scar is a long, raised slither of bone that extends dorsomedially down the posterior surface of the proximal portion of the femur. It trends from the lateral margin of the anterior trochanteric shelf to the dorsolateral margin of the fourth trochanter. It looks similar to the condition in *Asilisaurus kongwe* (NMT RB159). The posterior scar for linea intermuscularis caudalis is present and visible in all specimens except TMM 31100‐185. It is subtle in TMM 31100‐1309 and is obscured by breakage on TMM 31100‐1304. In TMM 31100‐1303, the scar for linea intermuscularis caudalis is a long groove that extends proximodistally on most of the posterolateral margin of the shaft of the femur. It closely resembles the condition observed in *Asilisaurus kongwe* (NMT RB159).

Distal portion: There is less obvious natural variation in the shape of the distal portions of each femur than in the proximal portions, but a great deal of variation in preservation and preparation. The posterior portion of TMM 31100‐1303 is the least damaged of all the specimens in terms of breakage and abrasion, but features a deep concavity covering the distalmost portion of the popliteal fossa on the posterior surface that is likely a pathology. The distal portion of TMM 31100‐1304 suffered breakage on the anterior surface and abrasion on the ventral and posterior surfaces (Figure 5). The surfaces of the distal portion of TMM 31100‐185 were smoothed artificially during preparation, obscuring many of the finer details, whereas TMM 31100‐1309 lacks the distal portion altogether.

The distal condyles do not mediolaterally expand much relative to the shaft of the femur. The medial condyle of TMM 31100‐185 and TMM 31100‐1304 extends further medially than in TMM 31100‐1303, though it is unclear the extent to which this is distorted by preservation. The sulcus forming the popliteal fossa is dorsoventrally long and deeper distally than proximally in TMM 31100‐185 and TMM 31100‐1304 (Figures 3 and 5). The shape of the posterior surface of the distal portion of the femur is similar to *Kwanasaurus williamparkeri* (e.g., DMNH EPV.67956; Martz & Small, 2019), *Asilisaurus kongwe* (e.g., NMT RB159), *Lewisuchus admixtus* (e.g., PULR V‐111; Agnolin et al., 2022), and *Silesaurus opolensis* (e.g., ZPAL AbIII/361/23; Piechowski et al., 2014).

On the distal surface of the femur, the anterior margin is gently rounded, whereas the lateral and medial margins are anteroposteriorly straight in all of the specimens. The medial condyle is a sub‐oval and projects far posteriorly to a pointed posterior margin. The medial condyle projects posteromedially (Figures 3 and 5) in TMM 31100‐185 and TMM 31100‐1304. In TMM 31100‐1303, the medial condyle projects posteriorly and slightly laterally, though this is possibly not natural, as with the popliteal fossa on this specimen (Figure 4). The lateral condyle, like in other non‐dinosaurian dinosauriforms (Nesbitt et al., 2020), is located along the anterolateral margin of the distal surface of the femur as a small, rounded projection. A prominent groove extends mediolaterally on the lateral part of the articular surface. The crista tibiofibularis is a small, posteriorly pointed projection that extends posterolaterally of the lateral condyle, and the posterior margin of the crista tibiofibularis is convex in distal view.

Collectively, the shape of the distal end of the Otis Chalk silesaurid most closely resembles *Silesaurus opolensis* (e.g., ZPAL Ab III/405) and *Sacisaurus agudoensis* (e.g., MCN PV10013; Langer & Ferigolo, 2013). Specifically, the shapes of the lateral condyle and crista tibiofibularis and the groove on the lateral part of the articular surface match that closely of the femur of *Kwanasaurus williamparkeri* (e.g., DMNH EPV.67956; Martz & Small, 2019).

### 6.3 Tibiae

**FIGURE 8:**
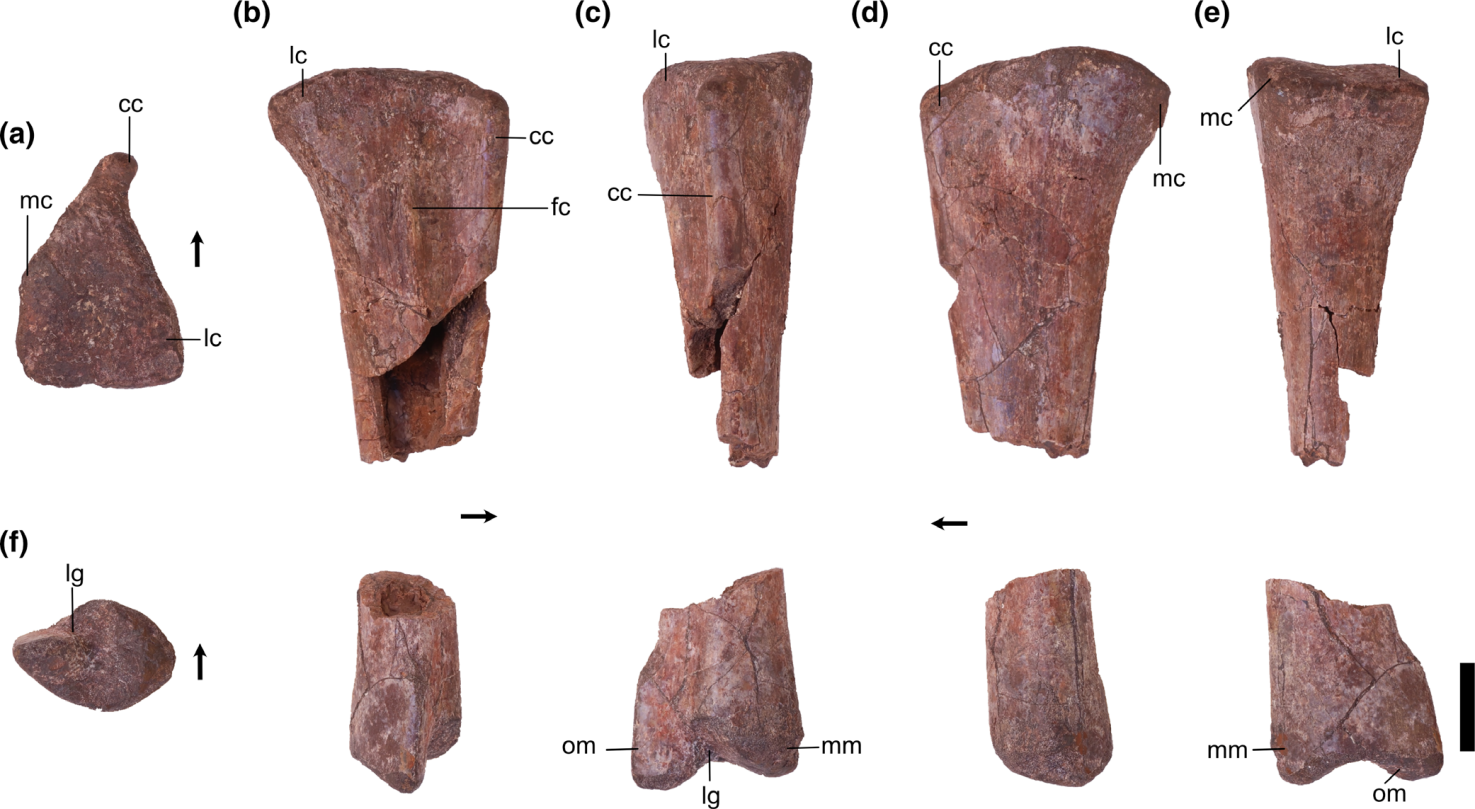
Silesauridae (TMM 31100‐1311) right tibia in proximal (a), lateral (b), anterior (c), medial (d), posterior (e), and distal (f) views. Cc, cnemial crest; fc, fibular crest; lc, lateral condyle; lg, lateral groove; mc, medial condyle; mm, medial malleolus; om, outer malleolus. Arrows represent anterior direction. Scale bar equals 1 cm.

**FIGURE 9:**
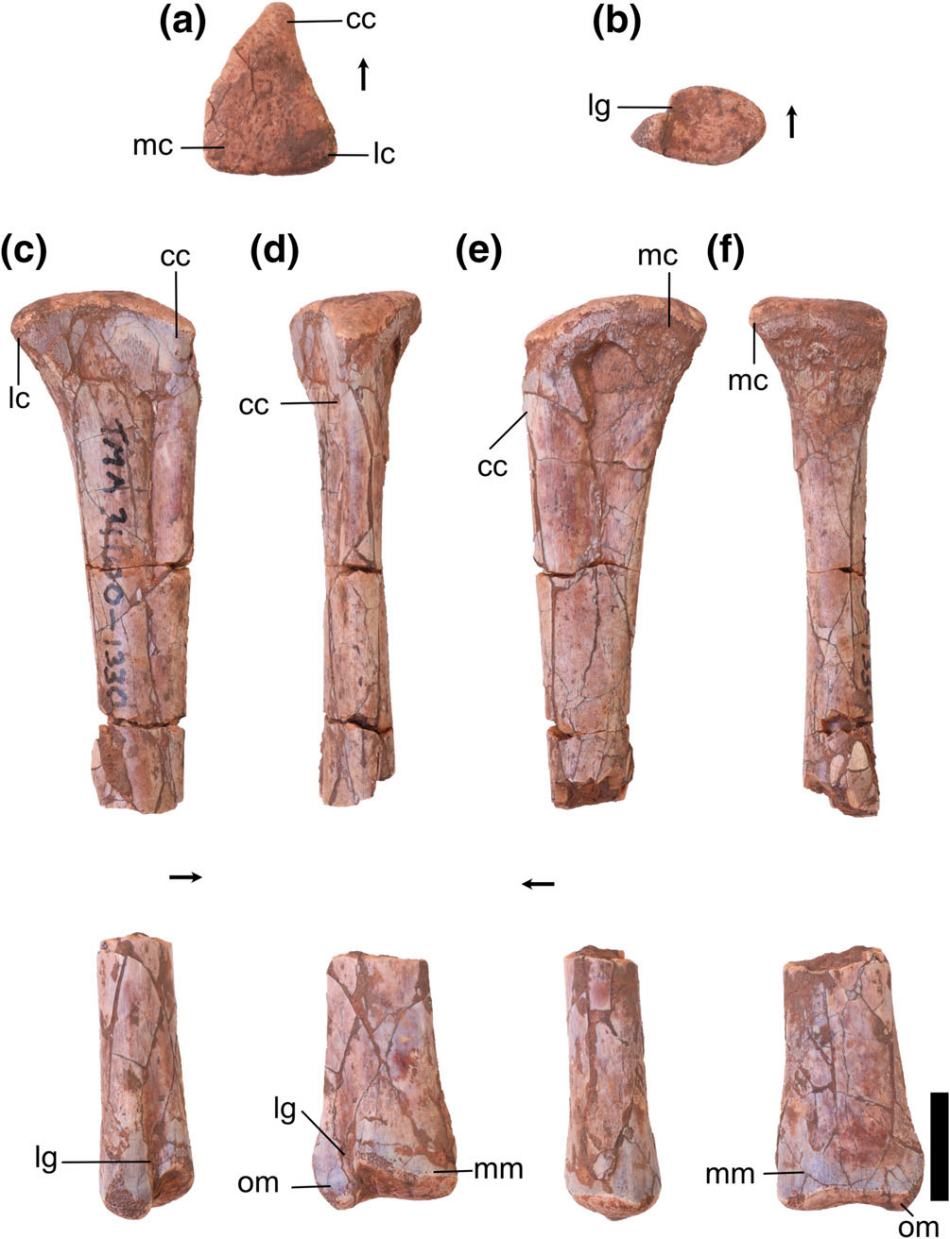
Silesauridae (TMM 31100‐1330) right tibia in proximal (a), distal (b), lateral (c), anterior (d), medial (e), and posterior (f) views. Cc, cnemial crest; lc, lateral condyle; lg, lateral groove; mc, medial condyle; mm, medial malleolus; om, outer malleolus. Arrows represent anterior direction. Scale bar equals 1 cm.

**FIGURE 10:**
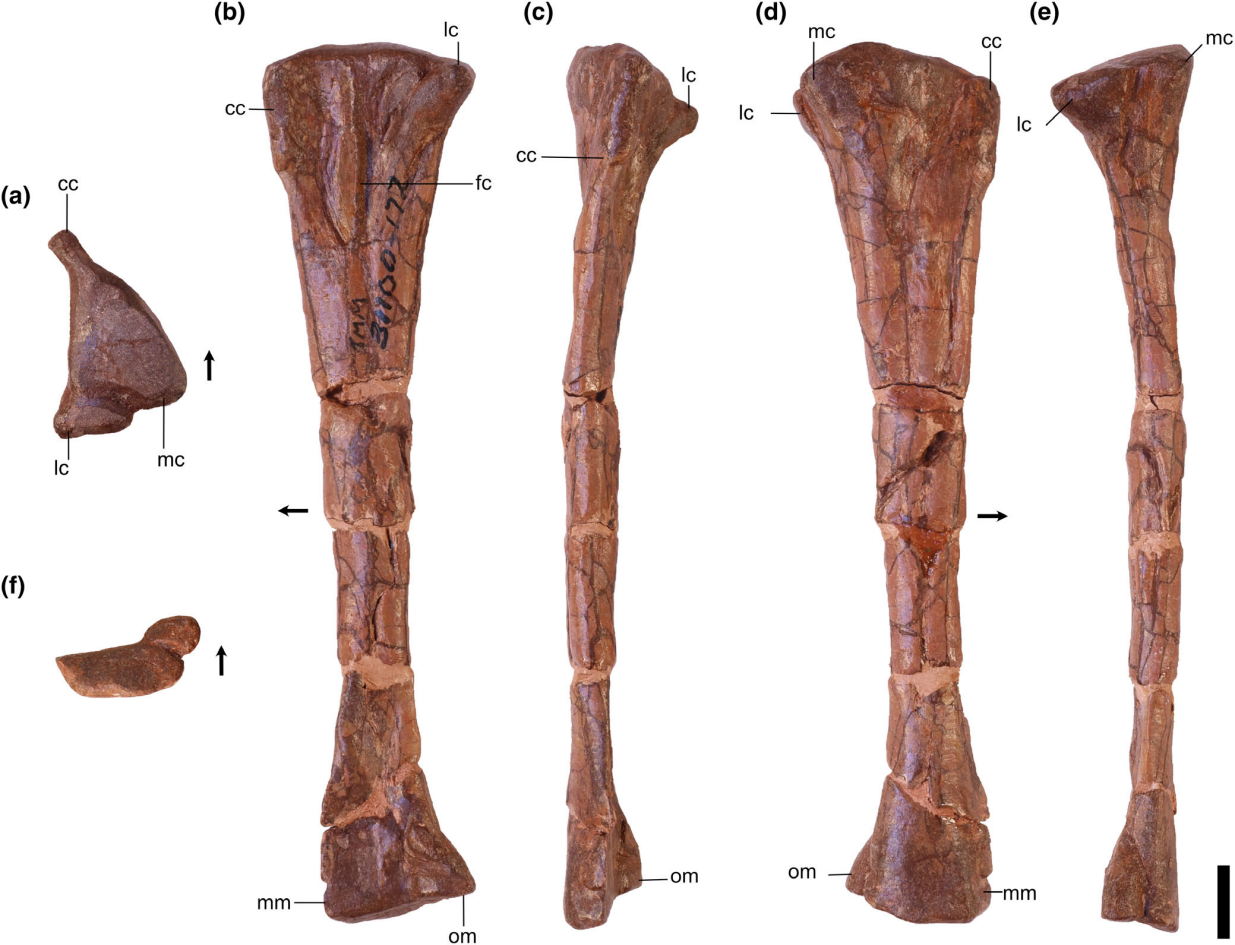
Silesauridae (TMM 311 00‐172) left tibia in proximal (a), lateral (b), anterior (c), medial (d), posterior (e), and distal (f) views. Cc, cnemial crest; fc, fibular crest; lc, lateral condyle; lg, lateral groove; mc, medial condyle; mm, medial malleolus; om, outer malleolus. Arrows represent anterior direction. Scale bar equals 1 cm.

**FIGURE 11:**
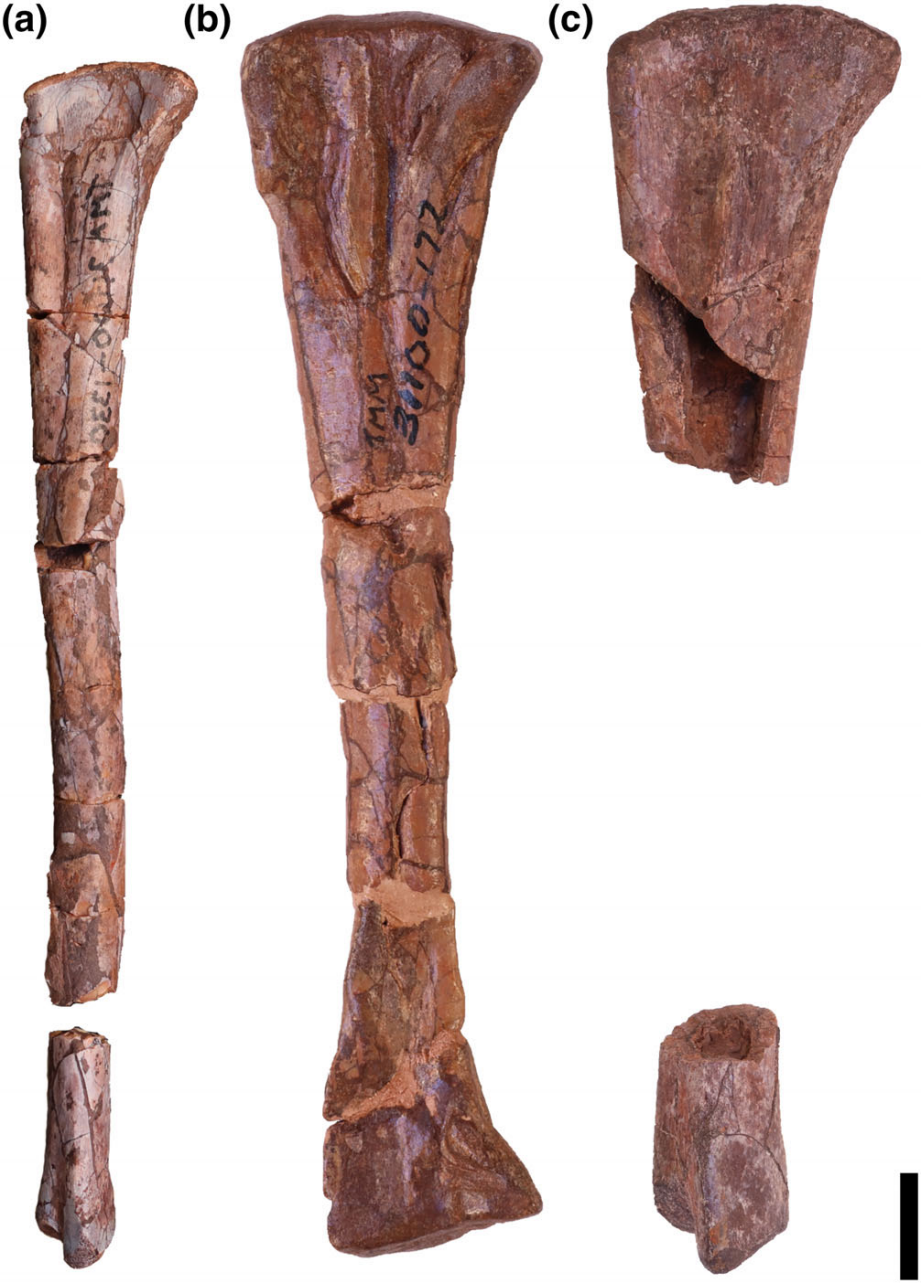
Silesauridae tibiae TMM 31100‐1330 (a), TMM 31100–172 (b), and TMM 31100–131 1 (c) in lateral view. (a) and (c) are mirrored. Scale bar equals 1 cm.

Overview: There are three tibiae identified (TMM 31100‐1311, Figure 8; TMM 31100‐1330, Figure 9; and TMM 31100‐172; Figure 11). TMM 31100‐1311 (Figure 8) includes complete proximal and distal ends of a right tibia and part of the shaft, though much of the shaft is missing. TMM 31100‐1330 (Figure 9) is a fragmentary right tibia consisting of a complete proximal and complete distal end, as well as most of the midshaft. TMM 31100‐172 (Figure 10) is a complete left tibia that sustained some breakage along the shaft and has some mediolaterally compression.

The tibiae are nearly completely straight dorsoventrally. The proximal portion is subtriangular in medial and lateral views and expands greatly beyond the width of the shaft. The distal portion is also subtriangular but distinctly smaller than the proximal portion. The overall shape in medial and lateral views is similar to *Sacisaurus agudoensis* (e.g., MCN PV10020; Langer & Ferigolo, 2013) and *Silesaurus opolensis* (e.g., ZPAL AbIII/361/22; Piechowski & Tałanda, 2020), and a number of dinosauriform specimens from some localities in the Eagle Basin Chinle (hereafter referred to as the “Eagle Basin Dinosauriformes”; DMNH EPV.56652, DMNH EPV.63875, DMNH EPV.67955; Martz & Small, 2019). The proximal and distal ends of TMM 31100‐172 flare mediolaterally to a greater extent than in *Asilisaurus kongwe* (NMT RB159) or *Lewisuchus admixtus* (e.g., PULR 01; Bittencourt et al., 2015). Overall, the specimens are quite similar to one another, except for that the proximal portion on TMM 31100‐1311 is less mediolaterally compressed (likely an artifact of preservation as previously mentioned), and TMM 31100‐1330 is long without much proximal and distal expansion compared with the other tibiae. Given other indications of TMM 31100‐1330 representing a younger individual than the other tibiae, we propose that this represents ontogenetic variation.

Proximal portion: The proximal surface of the tibia is subtriangular in proximal view with a cnemial crest that projects anteriorly and slightly laterally. The proximodistally long cnemial crest forms much of the anterior side of the proximal portion of the tibia. In proximal view, the parallel lateral and medial sides of the cnemial crest result in a mediolaterally ‘pinched’ shape in all three Otis Chalk specimens is not present in other silesaurids and likely represents an autapomorphy of the taxon represented by these specimens. The medial margin of the proximal surface is rounded posteriorly and tapers more sharply anteriorly towards the cnemial crest, though this anterior‐tapering is less‐pronounced in TMM 31100‐1330 (Figure 9). The lateral margin of the proximal surface, between the cnemial crest and the lateral condyle, is straight as the lateral and medial condyles are nearly aligned mediolaterally (though the former projects out a little further than the latter). This closely resembles the condition in the tibiae of *Sacisaurus agudoensis* (e.g., MCN PV10020; Langer & Ferigolo, 2013) and *Silesaurus opolensis* (e.g., ZPAL AbIII/361/22; Piechowski & Tałanda, 2020). It shares the same character scoring (character 331, state 1) in the character matrix of Nesbitt (2011) with the latter two taxa and some other non‐dinosaur dinosauriforms and early branching dinosaurs. In *Asilisaurus kongwe* (e.g., NMT RB159), and *Lewisuchus admixtus* (e.g., PULR V‐111; Agnolin et al., 2022) the lateral condyle is located more anteriorly on the lateral margin of the proximal surface. Both the overall shape of the cnemial crest and the alignment of the lateral and medial condyles in Otis Chalk specimens is similar to that of the Eagle Basin dinosauriforms (DMNH EPV.56652, DMNH EPV.63875, and DMNH EPV.67955; Martz & Small, 2019).

In medial view, the posterior half of the proximal margin of the tibia is convex, and the anterior half is straight and tapers distally. The proximal margin boasts two anteroposteriorly opposing suboval scars representing the hypothesized attachments for ligamentum collaterale tibiale (anterior) and m. puboischiotibialis + m. flexor tibialis internus + m. flexor tibialis externus (posterior) in *Silesaurus opolensis* (Piechowski & Tałanda, 2020). The latter continues along the area directly distal to the medial and lateral condyles on the posterior surface.

On the lateral surface of the tibia, the fibular crest extends dorsoventrally along the long axis of the femur (Figure 11). On the proximal portion of the tibia, the surface is concave anteriorly between the fibular crest and the cnemial crest, representing the attachment area for m. tibialis anterior (Griffin & Nesbitt, 2016a). On TM 31100‐172, the surface posterior to the proximal portion of the fibular crest is concave, but this was the result of crushing (Figure 10). The surface posterior to the proximal extent of the fibular crest represents the attachment area for m. popliteus (Piechowski, 2021). The anterior and posterior concave surfaces on either side of the fibular crest resemble the shape of the tibiae of *Sacisaurus agudoensis* (e.g., MCN PV10020; Langer & Ferigolo, 2013), *Silesaurus opolensis* (e.g., ZPAL AbIII/361/22; Piechowski & Tałanda, 2020), and the Eagle Basin dinosauriforms (e.g., DMNH EPV.63875; Martz & Small, 2019). In TMM 31100‐1330 (Figure 9), there is no fibular crest present on the lateral side of the proximal portion of the tibia, unlike in TMM 31100‐1311 (Figure 8), TMM 31100‐172 (Figure 10), *Silesaurus opolensis* (e.g., ZPAL AbIII/361/22; Dzik, 2003; Piechowski & Tałanda, 2020), and *Sacisaurus agudoensis* (e.g., MCN PV10020; Ferigolo & Langer, 2007). All specimens of *Asilisaurus kongwe* (e.g., NMT RB 159; Nesbitt et al., 2020) lack the crest. TMM 31100‐1330 features a small bone scar on the lateral side of the proximal portion, just distal to the proximal margin, likely representing the ligamentum tibio‐fibulare (as in Piechowski & Tałanda, 2020). The presence of a fibular crest in larger specimens but the lack of the same feature in smaller specimens within a potential ontogenetic series has not been documented in any other dinosauriform. This observation implies that the presence of a fibular crest, an often‐used phylogenetic character among dinosaurs and kin (e.g., Gauthier, 1986; Rauhut, 2003; Nesbitt, 2011; Baron et al., 2017), may be ontogenetically controlled in some dinosauriforms.

Distal portion: In medial and lateral view, the outer malleolus is a pointed subtriangular projection that extends more distally relative to the medial malleolus. On the distal portion of TMM 31100‐1330 (Figure 9), the outer malleolus does not appear as subtriangular in anterior view as in TMM 31100‐1311 (Figure 8), and TMM 31100‐172 (Figure 10) and is also smaller and does not flare as sharply laterally. The medial malleolus is a dorsoventrally shorter subtriangular projection in medial view. In lateral view, the anterior margin of the medial malleolus is convex and is formed by the surface with which the ascending process of the astragalus articulates with the tibia. The medial malleolus flares medially to a slightly greater extent in TMM 31100‐1330 than in TMM 31100‐1311 (Figure 11).

On the distal surface of the tibia, the articular surface with the ascending process of the astragalus extends from the anteroposterior middle of the bone creating a ‘horseshoe’ shaped medial portion of the distal surface of the tibia. The shape of the distal portion of the tibia is similar to those of *Sacisaurus agudoensis* (e.g., MCN PV10020; Langer & Ferigolo, 2013), *Silesaurus opolensis* (e.g., ZPAL AbIII/361/22; Piechowski & Tałanda, 2020), and the Eagle Basin dinosauriforms (e.g., DMNH EPV.63875; Martz & Small, 2019). The distal margin of the tibia of *Asilisaurus kongwe* (e.g., NMT RB159), by contrast, is nearly flat. The groove on the articular surface with the ascending process of the astragalus of TMM 31100‐1330 is not as deep as in TMM 31100‐1311, and therefore the distal margin of the tibia is not as concave in anterior view. This condition is more similar to *Asilisaurus kongwe*, which has a flatter distal articular surface (e.g., NMT RB 159; Nesbitt et al., 2019).

## 7 PHYLOGENETIC ANALYSIS

**FIGURE 12:**
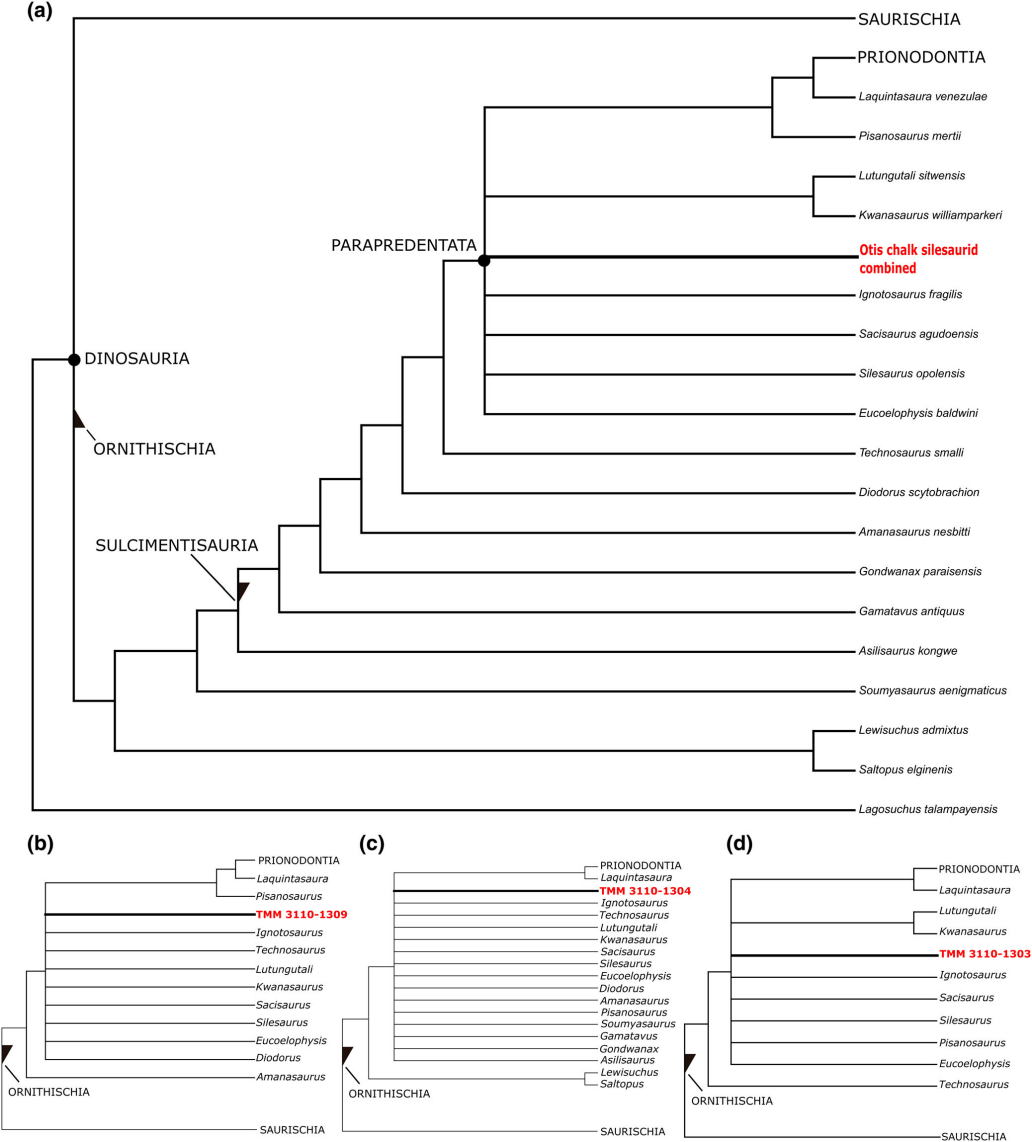
Results of phylogenetic analyses. (a) Strict consensus tree for the analysis treating TMM 31100‐1303 (femur), TMM 31100‐1330 (tibia), and TMM 31100‐1319 (humerus) as a combined OTU. (b) Strict consensus tree for the analysis including only TMM 31100‐1309 (femur with lowest OMS value, Table 1). (c) Strict consensus tree for the analysis including only TMM 31100‐1304 (femur with next‐lowest OMS value, Table 1). (d) Strict consensus tree for the analysis including only TMM 31100‐1303 (femur with highest OMS value, Table 1).

The analysis using the combined OTU returned 1008 most parsimonious trees (MPTs) and recovered silesaurids as a paraphyletic grade at the base of Ornithischia (Figure 12a), consistent with previous iterations of this matrix (Müller, 2025; Norman et al., 2022). The Otis Chalk silesaurid was recovered as a later‐branching member of this grade with affinities within Parapredentata (Norman et al., 2022). The combined OTU forms part of a polytomy alongside *Ignotosaurus fragilis*, *Sacisaurus agudoensis*, *Silesaurus opolensis*, *Eucoelophysis baldwini*, and the node (*Kwanasaurus williamparkeri* + *Lutungutali sitwensis*). This polytomy is sister to the clade including *Pisanosaurus mertii* and all other ornithischians (Figure 12.A). The node including the Otis Chalk silesaurid is supported by a single character: Dentary; Mandibular buccal emargination (Character 77.1: Present). This character state is unknown in the Otis Chalk silesaurid.

The analysis for TMM 31100‐1309, the smallest femur, returned 6192 MPTs. TMM 31100‐1309 is recovered within Sulcimentisaura, though not within Parapredentata in the strict consensus (Figure 12b). As with the combined OTU, TMM 31100‐1309 is part of a polytomy that is expanded to include *Diodorus scytobrachion* and *Technosaurus smalli*. *Lutungutali sitwensis* and *Kwanasaurus williamparkeri* are also no longer recovered as sister taxa. The most inclusive clade including TMM 31100‐1309 is supported by a single character: Femur; head, facies articularis antitrochanterica present (Character 216.0: level with “greater trochanter”).

The analysis for TMM 31100‐1304, a mature femur, returned 2160 MPTs. As with the analysis for TMM 31100‐1309, the polytomy of which TMM 31100–1304 is part has expanded to include all earlier‐branching ornithischians except the node (*Lewisuchus admixtus* + *Saltopus elginenis*; Figure 12c). Furthermore, *Pisanosaurus mertii* is now also part of this polytomy, with *Laquintasaura venezulae* recovered as the earliest‐branching member of the clade including all later‐diverging ornithischian taxa.

The analysis for TMM 31100‐1303, the most mature femur, recovered 1296 MPTs. TMM 31100‐1303 forms part of a polytomy identical to that in the analysis using the combined OTU but except that it also includes *Pisanosaurus mertii*. *Laquintasaura venezulae* is, once again, recovered as the earliest‐branching member of the clade including all later‐diverging ornithischian taxa (Figure 12d).

## 8 DISCUSSION

The Otis Chalk silesaurids add an important dimension to our understanding of the North American fossil record and the broader evolutionary success of this group throughout the Triassic. Silesaurids are present throughout the Dockum Group and Chinle Formation (Marsh & Parker, 2020) but are exceedingly rare in the lower parts of those strata. Our study presents the first definitive record of silesaurids from the Otis Chalk localities and, as such, one of the older known fossil records of silesaurids from the Dockum Group and Chinle Formation (Lucas et al., 1993; Stocker, 2013). Currently, the oldest known North American record of silesaurids is from the mid‐to‐late Carnian‐aged Popo Agie strata (Lovelace et al., 2025), but there is no younger record in the same area. Conversely, the addition of the specimens from the Otis Chalk to the existing silesaurid record from the Dockum Group demonstrates the presence of silesaurids from the late Carnian or early Norian (see above) and indicates a continued presence of this group throughout the Late Triassic. Integrating the Dockum Group and Chinle Formation records with that of other occurrences in North America, silesaurids were present well into the late Norian or into the Rhaetian (Marsh & Parker, 2020). Further combined with older records of the group from southern Pangean deposits (Müller & Garcia, 2023; Nesbitt et al., 2010) in the Middle and Upper Triassic, silesaurids were clearly a widespread and long‐lived group across the Triassic Period.

The Otis Chalk silesaurid is recovered in phylogenetic analysis as a member of the paraphyletic grade including taxa traditionally included in monophyletic Silesauridae hypotheses (e.g., Nesbitt et al., 2010). More precisely, the Otis chalk silesaurid taxon is of close phylogenetic affinity with *Silesaurus opolensis*, *Eucoelophysis baldwini*, and other early‐branching members of Parapredentata following the phylogenetic hypothesis of Norman et al. (2022). This small sample of silesaurids from the Otis Chalk also demonstrates the high variability in certain morphological features of the femora similar to other dinosauriforms. The description above shows that the femora vary in both length and the presence of features associated with growth in dinosauriforms (see Griffin & Nesbitt, 2016a, 2016b). The two femora displaying lower observed maturity score values (see Table 1) are recovered in less nested positions within the grade when they are scored separately into a phylogenetic analysis, consistent with the results of Müller et al. (2024). To investigate the combination of length and character states associated with growth, we took two approaches. First, we conducted a traditional cladistic ontogeny method (Brochu, 1992) and second, we compared reconstructed sequence polymorphism (e.g., Ontogenetic sequence analysis; Colbert, 1999; Colbert & Rowe, 2008) of closely related taxa.

Our cladistic ontogeny method, implemented in PAUP (v. 4.0b10; Swofford, 2002), revealed some information regarding the relative maturity of each specimen. Our analysis resulted in two most parsimonious trees (MPTs). The strict consensus resulted in a polytomy between the three more‐mature semaphoronts. TMM 31100‐1309 was found as the sister taxon of the others; therefore, it was found as the least mature semaphoront. The other three specimens were recovered in a polytomy. This method resulted in a similar ontogenetic stage for TMM 31100‐185, TMM 31100‐1303, and TMM 31100‐1304. However, the highly modified surfaces of TMM 31100‐185 during preparation did not allow us to score many of the character states, so this result is not conclusive for this individual, and its removal from the analysis would leave only a single possible topology (TMM 31100‐1309(TMM 31100‐1303 + TMM 31100‐1304)). Our cladistic ontogeny analysis also could not differentiate TMM 31100‐1303 and TMM 31100‐1304 because they had a similar set of scores, but conflicting character states do not inform which specimen is most mature. The lack of resolution of the maturity among these specimens and other confounding factors (e.g., loss of information because of preparation process) led us to use our second method.

We also compared the combinations of features of the Otis Chalk femora to those of the larger sample of *Asilisaurus kongwe*, a closely related silesaurid that was analyzed using ontogenetic sequence analysis (Griffin & Nesbitt, 2016b). The low sample size of the Otis Chalk femora did not allow us to reconstruct the sequence polymorphism, but we were able to score the same characters and directly compare the combination of growth features of each femur with that of the reconstructed sequence of less mature to more mature individual combinations of *Asilisaurus kongwe*. To estimate the possible ontogenetic stages of the femora of the Otis Chalk silesaurids, we mapped each specimen's features on the ontogenetic sequence figure presented in Griffin & Nesbitt (2016a; Figure 7). At an estimated 98 mm in length (Table 2), TMM 31100‐1309 represents the smallest individual in the sample. As would be expected, the proximal portion of TMM 31100‐1309 preserves the fewest muscle attachment sites identified by Griffin and Nesbitt (2016a) (Table 2). Hence, TMM 31100–1309 represents the least mature specimen of this silesaurid assemblage.

The other two better preserved femora represent more mature individuals (TMM 31100‐185 was excluded because of lack of character scores, see above). It is likely that they were at similar stages of ontogenetic development but exhibit intraspecific developmental polymorphism similar to that estimated for *Asilisaurus kongwe* (Griffin & Nesbitt, 2016a). These two specimens (TMM 31100‐1303, TMM 31100‐1304) are of similar length (152 mm, and 155 mm, respectively) but possess a different set of growth character states associated with maturity. For example, the anterior trochanter and the trochanteric shelf (“at+ts” of Griffin & Nesbitt, 2016a) are fused in TMM 31100‐1303 but not in TMM 31100‐1304, suggesting the former represents a more mature specimen (Griffin & Nesbitt, 2016a). Despite the anterior trochanter and the trochanteric shelf being scored as absent in TMM 31100‐1304, it has a thicker femoral circumference (52 mm) than that of TMM 31100‐1303 (43 mm), suggesting it represents a heavier individual (Campione & Evans, 2012). On the other hand, there is no M. *caudofemoralis brevis* insertion scar present on the posterolateral edge of TMM 31100‐1303, a character state correlated with later maturity in *Asilisaurus kongwe* (Griffin & Nesbitt, 2016a). An M. *caudofemoralis brevis* insertion scar is present on the posterolateral edge of TMM 31100‐1304. Using this growth character, TMM 31100‐1303 represents a less mature individual than TMM 31100‐1304. TMM 31100‐1303, and TMM 31100‐1304 have more mature character scores than that of TMM 31100‐1309, but the combination of those character scores is not the same. Hence, we conclude that the Otis Chalk silesaurid possessed similar sequence polymorphism as its close relative *Asilisaurus kongwe*.

From our description and set of analyses, alongside our consultation of the existing literature, we hypothesize that the variation in morphology between the Otis Chalk femora is attributable to ontogenetic variation in a growth series of a single silesaurid taxon. We cannot fully rule out that there is more than one species of silesaurid at the locality, but the recorded sample from the locality matches the variation in other silesaurids (e.g., *Asilisaurus kongwe*, Griffin & Nesbitt, 2016a; *Silesaurus opolensis*, Piechowski et al., 2014), early‐diverging sauropodomorph *Saturnalia tupiniquim* (Damke et al., 2024), and early‐diverging neotheropod dinosaurs *Megapnosaurus rhodesiensis* (Griffin, 2018) and *Coelophysis bauri* (Griffin, 2018; Griffin & Nesbitt, 2016b; Griffin et al., 2021). We also propose two ontogenetic changes in tibia shape from this sample: greater expansion of the proximal and distal ends in adults and a lack of fibular crest in juveniles. The combination of size and growth feature differences in the same element (e.g., tibiae and femora) is considerable even in this small sample, and we urge against naming new taxa from the same locality based on characters correlated with growth. Lastly, our study highlights the importance of interpreting taphonomic preservation and preparation quality in assessing growth features. Accurate description and scoring of growth character states require well‐preserved surfaces that are well prepared. If the specimens are distorted or features have been removed through preparation, analyses may be predicting lower maturities or inflated polymorphism. This, furthermore, may affect taxonomic diagnoses, interpretation of palaeobiological traits, growth reconstruction, and reconstructions of palaeoecology.

## AUTHOR CONTRIBUTIONS

**Frederick B. Tolchard:** Investigation; writing – original draft; methodology; formal analysis; software; writing – review and editing; data curation; conceptualization; validation; visualization. **Brynden W. Perkins:** Writing – review and editing; visualization; validation; data curation; investigation. **Sterling J. Nesbitt:** Supervision; data curation; resources; funding acquisition; conceptualization; investigation; project administration; writing – review and editing; validation; methodology.

## FUNDING INFORMATION

This study was funded by National Science Foundation (EAR 1943286) to S. J. N. National Research Foundation (NRF) of South Africa (NRF AOP 136516 and MND200718544868).

## CONFLICT OF INTEREST STATEMENT

The authors declare no conflicts of interest.

## Supporting information


**APPENDIX S1:** Supporting information.
